# Carbonized Plant Powder Gel for Rapid Hemostasis and Sterilization in Regard to Irregular Wounds

**DOI:** 10.3390/nano14241992

**Published:** 2024-12-12

**Authors:** Zhong Liu, Shaolei Ding, Guodong Zhang, Bingyu Yan, Chao Zhang, Pihang Yu, Yunze Long, Jun Zhang

**Affiliations:** Collaborative Innovation Center for Nanomaterials & Devices, College of Physics, Qingdao University, Qingdao 266071, China

**Keywords:** carbonized plant powder, gel, rapid hemostasis, photothermal stabilization, irregular wounds

## Abstract

Irregularly shaped wounds cause severe chronic infections, which have attracted worldwide attention due to their high prevalence and poor treatment outcomes. In this study, we designed a new composite functional dressing consisting of traditional Chinese herb carbonized plant powder (CPP) and a polyacrylic acid (PAA)/polyethylenimine (PEI) gel. The rapid gelation of the dressing within 6–8 s allowed the gel to be firmly attached to an irregularly shaped wound surface and avoided powder detachment. In addition, through an infrared thermography analysis, a coagulation assay, and a morphological examination of regenerative tissue in animal wound models, it was found that the dressing substrates had synergistic effects on photothermal sterilization, rapid hemostasis, and anti-inflammatory activity, thereby achieving an 88% wound closure rate on the 9th day after the formation of the wound. This multifunctional hemostatic material is expected to be adaptable to irregular wounds and promote rapid wound healing.

## 1. Introduction

The emergency treatment of wounds in daily life is crucial, and it has been suggested that hemostasis and antibacterial measures are the two most critical factors [1,2]. However, due to the irregular shapes of some wounds, dressings are often unable to fully cover them. The presence of gaps between the dressing and the wound increases the risk of bacterial infection and compromises the hemostatic effect, leading to delayed wound healing [3]. Generally, hemostasis is achieved by pressing the dressing onto the surface of the wound. This simple approach results in unsatisfactory hemostatic effects. While traditional dressings serve to isolate wounds, the large pores in medical gauze contribute to wound infection issues [4,5]. Modern moist interactive dressings such as hydrogels, foams, films, topical antibiotics, and calcium alginates [6] are widely employed, but their limitations make it difficult to adapt them to complex wounds, especially the narrow gaps of deep wounds [7]. Therefore, there is an urgent need to develop a hemostatic material that can adapt to irregular wounds and promote effective wound healing.

Traditional Chinese medicine carbonized plant powder (CPP) consists of the black soot residue that forms at the bottom of a pot and chimney after burning straw and wheat straw, commonly known as soot from the bottom of a boiler. Research records indicate that CPP possesses hemostatic, sterilizing, and anti-inflammatory properties. The phenolic compounds in CPP have been found to inhibit platelet aggregation and facilitate the release of coagulation factors, thereby effectively promoting hemostasis [8,9]. However, due to its hydrophobic characteristic, CPP exhibits poor tissue adhesion and can easily be washed away by blood from wound surfaces, resulting in suboptimal coagulation effects [10,11]. To address this issue, hydrogel, a novel polymer material with a unique three-dimensional network structure and high water content, can be utilized, and it has been widely applied in wound-dressing applications [12]. Hydrogel synthesized from polyacrylic acid (PAA) and polyethyleneimine (PEI) demonstrates excellent water absorption capabilities. After being freeze-dried and ground into powder form [13,14], it rapidly absorbs interfacial water near skin tissues upon contact with wounds to form physically cross-linked hydrogels [15,16,17]. Furthermore, the cross-linked polymers can infiltrate the substrate polymer network and thus provide strong tissue adhesion. Additionally, this hydrogel powder can undergo robust self-healing upon water absorption and thus revert to its original gel state [18,19], enabling superior adaptation to various irregularly shaped wounds for optimal wound-fitting purposes [20,21]. Moreover, owing to its exceptional adhesion and hydrophilicity, hydrogel powder can absorb surrounding blood rapidly and then induce physical hemostasis [22,23]. However, PAA/PEI powder lacks photothermal antibacterial ability [24]. Photothermal therapy, a popular physical method in the field of antibacterials in recent years and characterized by excellent antibacterial ability against a variety of bacteria, can induce bacterial death in a short time after absorbing light, and it is safer than using chemical antibacterials [25]. Therefore, adding functional materials and addressing the shortcomings of PAA/PEI through photothermal antibacterial treatment are highly innovative approaches.

In this study, Chinese herbal medicine CPP was mixed into PAA/PEI hydrogel as a carrier for wound dressings. After being freeze-dried and ground, PAA/PEI/CPP powder was obtained. Once the powder is gelatinized, it not only prevents CPP from falling off easily but also combines the physical hemostasis mechanism of the hydrogel with the biochemical hemostasis mechanism of CPP to achieve synergistic effects and promote hemostasis effectively. Compared with traditional dry wound dressings, a moist environment is more conducive to irregular wound recovery. Additionally, it has photothermal sterilization, hemostasis promotion, and anti-inflammatory effects. The physicochemical properties of gel powder and its abilities to resist bacteria through photothermal effects and promote wound healing fully validate the potential of CPP composite gel as an agent for achieving hemostasis.

## 2. Experimental Section

### 2.1. Materials

Poly (acrylic acid) aqueous solution (PAA, Mw ≈ 240,000, 25 wt%) was purchased from J&K Scientific (Beijing, China). Polyethyleneimine aqueous solution (PEI, Mw ≈ 70,000, 50 wt%) was purchased from Macklin (Shanghai, China). Carbonized plant powder (CPP) was purchased from the Pharmacy Department, the Affiliated Hospital of Qingdao University.

### 2.2. Synthesis of PAA/PEI/CPP Powder

First, 0.5 g of CPP was added to 4.5 g of deionized water, stirred evenly at 24 °C to form a suspension of pennyroyal cream, mixed with 15 g of PEI aqueous solution, and then stirred for 6 h at 24 °C. Subsequently, the CPP/PEI aqueous solution was blended with 15 g of PAA aqueous solution to prepare PAA/PEI/CPP gel. The PAA/PEI/CPP gel was quickly frozen in liquid nitrogen. After being freeze-dried and ground, with an approximate mass ratio of 7.5:15:1, PAA/PEI/CPP powder was obtained.

### 2.3. Material Characterization

A scanning electron microscope (SEM, Phenom Pro, Phenom, Eindhoven, The Netherlands) was used to observe the microscopic structures of the hydrogels. A Phenom Pro scanning electron microscope was applied to analyze the microscopic morphology of the samples, including the shapes of particles and the internal structure and pore distribution of the samples.

A Fourier infrared spectrometer (Nicolet iS50, Thermo Fisher Scientific, Waltham, MA, USA) was adopted to analyze the different optical paths generated by the interference of infrared light so as to conduct qualitative and quantitative analyses of the chemical composition and structure of the material. The wavelength ranged from 2000 to 500 cm^−1^, with a 4 cm^−1^ resolution and a total of 32 Fourier transform infrared scans.

A water absorption rate test was conducted. The PAA/PEI and PAA/PEI/CPP powders were immersed in deionized water at 37 °C for 24 h and then freeze-dried for 48 h to remove the water, and the mass of the dried samples was recorded as M_0_. Subsequently, the dried samples were immersed in sufficient deionized water at 37 °C and then weighed after absorbing water at 0.5 h, 1.5 h, 6 h, 12 h, 24 h, and 72 h, and the mass of the samples after water absorption was recorded as M_1_. All experiments were repeated three times. The water absorption rate was calculated as follows: water absorption rate = (M_1_ − M_0_)/M_0_ × 100% [24].

A tensile test was conducted using a universal material testing machine (5300, 50 N load cell, Instron, Norwood, MA, USA). The PAA/PEI hydrogel and PAA/PEI/CPP gel were clamped in the flat chuck. The tensile parameters were as follows: the distance between the upper and lower chucks was d, and the tensile speed was 50 mm/min. The calculation of tensile strength was as follows: tensile strength = F/s × h, where F is the stress, and s and d are the width and thickness of the samples, respectively.

A discovery hybrid rheometer (DHR3, TA Instruments, New Castle, DE, USA) was used in a shock scanning experiment, which was conducted at 37 °C, 1% strain, and 1 Hz. In a frequency sweep experiment, the frequency was swept from 10 Hz to 0.1 Hz. The storage modulus and loss modulus of the samples before and after gelation were measured to analyze their properties.

### 2.4. Tissue Adhesion Experiment

The PAA/PEI/CPP powder was tiled in a middle piece of pig skin with a 10 cm length, 2 cm width, and 0.2 cm thickness, and then the pig skin was rotated to verify the adhesion of the PAA/PEI/CPP powder to the skin tissue. Additionally, the adhesion of the PAA/PEI/CPP powder was also tested on multiple materials. Two glass plates (2 cm × 2 cm) were fixed together using the powder, with a 20 g weight suspended beneath them. Comparably, a piece of pig skin with a 10 cm length, 2 cm width, and 0.2 cm thickness was attached to one glass plate (2 cm × 2 cm) with a 20 g weight hung below it.

### 2.5. Fibrocyte Compatibility Experiment

The samples were divided into three groups: a control group, PAA/PEI powder group, and PAA/PEI/CPP powder group. Firstly, the samples were placed onto a 60 mm sterile culture dish, then 1 mL PBS was added for washing twice, and 1 mL complete culture medium was blended as a standby. Secondly, the fibroblasts were removed from the incubator, washed three times with PBS, and then digested with 1 mL trypsin on the culture dish. After two minutes, digestion was terminated with 1 mL complete medium. Lastly, the samples were centrifuged in a 5 mL EP tube at 1200 rmp for five minutes, the supernatant was poured out, and the cells were resuspended with 1 mL complete medium. Then, the cell suspension was injected onto the sterile culture dishes of the blank group and the experimental group separately, and well mixed. A laser confocal microscope (AX, Nikon, Kanagawa, Japan) was used to observe live cells.

### 2.6. Antibacterial Experimentation

Initially, 20 µL of *E. coli* and 20 µL of *S. aureus* were inoculated onto agar medium (24102402, Bena Culture Collection, Suzhou, China) and evenly spread using a sterile smear stick. Subsequently, the prepared samples were placed at the center of the agar in the experimental group and exposed to 808-nanometer laser irradiation using a thermal infrared imager (246 M, FOTRIC, Shanghai, China), while no treatment was applied to the control group. Finally, all plates were cultivated at a constant temperature of 37 °C. After 12 h of cultivation, the plates were removed from the culture medium and photographed for observation.

On the 3rd, 5th, 7th, and 9th days, sterile cotton swabs were used to wipe the surface of the wound and collect samples. Then, the samples were inoculated onto agar plates and incubated at 37 °C for 24 h. Bacterial growth was observed, with Image J software (version number: 1.53t) used to count the number of colonies.

### 2.7. Coagulation Assay

Next, 30 mg of PAA/PEI powder, 30 mg of CPP, and 30 mg of PAA/PEI/CPP powder were placed onto the 96-well plates. Correspondingly, the control group served as blank wells. Then, 2 g anhydrous calcium chloride was dissolved in 10 g rabbit blood, containing sodium citrate anticoagulant, and stirred for 3 min. Subsequently, 100 µL of the rabbit blood was added to both the control group and experimental group wells. At pre-determined observation time points, the uncoagulated blood was thoroughly washed with PBS buffer solution. Finally, the coagulation of blood on each well plate was observed.

The PAA/PEI powder, CPP, and PAA/PEI/CPP powder were placed into centrifuge tubes, followed by the addition of 100 µL anticoagulated rabbit blood and 10 µL, 0.2 M calcium chloride solution. After standing for 5 min, 1 mL of PBS buffer solution was introduced into each centrifuge tube. Finally, the tubes were inverted to observe the formation of blood clots at the top.

### 2.8. Recovery Experiment of Animal Wounds

To validate the therapeutic efficacy of the various materials on traumatic wounds, 20 8-week-old male Sprague Dawley (SD) rats were purchased from Jinan Pengyue Experimental Animal Breeding Co., Ltd., Jinan, China. Ethical approval was obtained from Qingdao University Laboratory Animal Welfare Ethics Committee (No. 20230620SD3220230708019). Two circular wounds measuring 15 mm in diameter were produced on the dorsal region of the rats. They were divided into four groups, and the experimental groups consisted of three treatment cohorts, namely, CPP (Light-) treatment, PAA/PEI powder treatment, and PAA/PEI/CPP powder treatment, with the treatments applied directly onto the wound surface. The remaining group was treated as a blank control group. The wound surface was captured through photographic documentation on the 3rd, 5th, 7th, and 9th days to monitor the progression of wound healing using Image J software (version number: 1.53t).

### 2.9. Morphological Examination of Regenerative Tissue

The regenerating tissues from the wound in the dorsal region of the rats were collected on the 3rd and 9th days. Subsequently, they were fixed with a solution containing 4% paraformaldehyde. After fixation, the tissues were embedded in paraffin and cross-sectioned into slices with a thickness of 4 µm. These tissue sections were then subjected to staining using H&E and Masson trichrome staining kits. Finally, an inverted fluorescence microscope (IX53, Olympus, Tokyo, Japan) was used to analyze the pathological sections, following the manufacturer’s instructions.

### 2.10. Statistical Analysis

All results were obtained from at least three independent replicates and were expressed as mean value ± SD. A one-way analysis of variance was used for a statistical analysis, and a *p* value of less than 0.05 was considered statistically significant (* *p*  <  0.05, ** *p* <  0.01, *** *p* <  0.001).

## 3. Results

### 3.1. Morphological Characteristics and Properties

**Figure 1:**
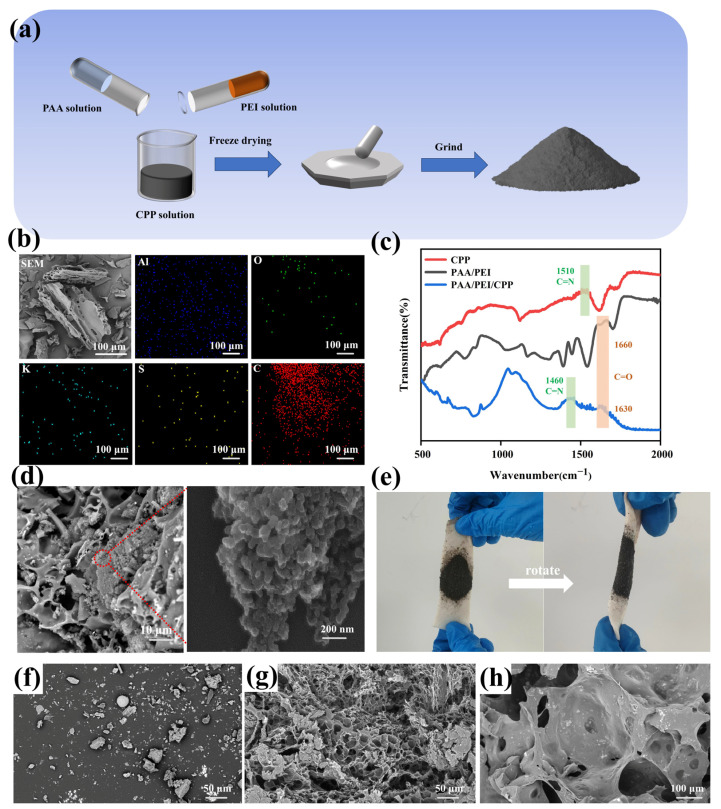
(**a**) Schematic diagram of the preparation process of PAA/PEI/CPP powder. (**b**) SEM image of the powder and analysis image of Al, O, K, S, and C elements. (**c**) FTIR spectra of CPP, PAA/PEI powder, and PAA/PEI/CPP powder. (**d**) SEM image of the gel powder and local enlargement. (**e**) Adhesion test of PAA/PEI/CPP powder on pig skin and between different materials. (**f**) CPP particles before being ground. (**g**) Freeze-dried PAA/PEI hydrogel powder before being ground. (**h**) Freeze-dried PAA/PEI/CPP gel powder before being ground.

Figure 1a shows a schematic diagram of the PAA/PEI/CPP powder synthesis process. The PAA and PEI aqueous solutions were combined in equal proportions, followed by the addition of the CPP suspension to generate PAA/PEI/CPP gel. Subsequently, freeze drying and grinding were carried out to acquire the gel powder. During the material preparation process, it was noted that the PEI aqueous solution carried a dense positive charge, which might have an adverse impact on cell membranes. To avoid this situation as much as possible, the equivalent amount of PAA aqueous solution was added, and this reduced the charge of the PEI solution. Figure 1b shows an elemental analysis (EDS) diagram of the hydrogel powder. The EDS diagram indicates that Al, O, K, S, and C were mainly present in the hydrogel powder and evenly distributed in the PAA/PEI/CPP gel, among which Al, S, and K were from the CPP; this means that the CPP soot was formed by rice straw, wheat straw, and other materials adhering to the bottom of the pot after combustion. Further infrared characterization of the gel powder was carried out, as shown in Figure 1c. After combustion, the CPP produced nitrogenous compounds, which was also one of the reasons why it became alkaline. For this reason, an infrared test found that there was a stretching vibration peak near 1500 cm^−1^, corresponding to the C=N group at 1510 cm^−1^ in the CPP, and it shifted to 1460 cm^−1^ in the PAA/PEI/CPP gel. Moreover, the C=O group at 1660 cm^−1^ in PAA/PEI shifted to 1630 cm^−1^ in the PAA/PEI/CPP gel, indicating that both of the chemical bonds shifted after being combined with the hydrogel. Figure 1d presents a locally enlarged SEM image of PAA/PEI/CPP, in which it can be seen that CPP particles were embedded in the dense porous structure after being ground; additionally, the grinding process contributed to a minimal damage to the porous structure. For the sake of a visual adhesion test, Figure 1e displays a gel powder coating on a wet pig skin surface, which rapidly coagulated into gel after contacting with moisture and adhered to the pig skin surface. Subsequently, the pig skin was rotated by 180°, and no shedding signs were observed, indicating that CPP exhibits strong adhesion to wet tissue (e.g., the pig skin surface) when combined with hydrogel. Furthermore, the adhesion of the PAA/PEI/CPP gel to diverse materials was investigated (Figure S1). The PAA/PEI/CPP gel was adopted to place in contact with the interface between two glass plates and the interface between glass and pig skin, with a 20 g weight suspended below. No observable detachment between the glass plates or between the pig skin and the glass plate was evident, attesting to the outstanding adhesion of the PAA/PEI/CPP gel to various dissimilar interfacial materials. In order to visually compare the morphology, Figure 1f indicates that, before being ground, the CPP particles were coarse and irregular in shape. Figure 1g shows the dense porous structure of PAA/PEI. Figure 1h shows the freeze-dried PAA/PEI/CPP gel powder before being ground.

**Figure 2:**
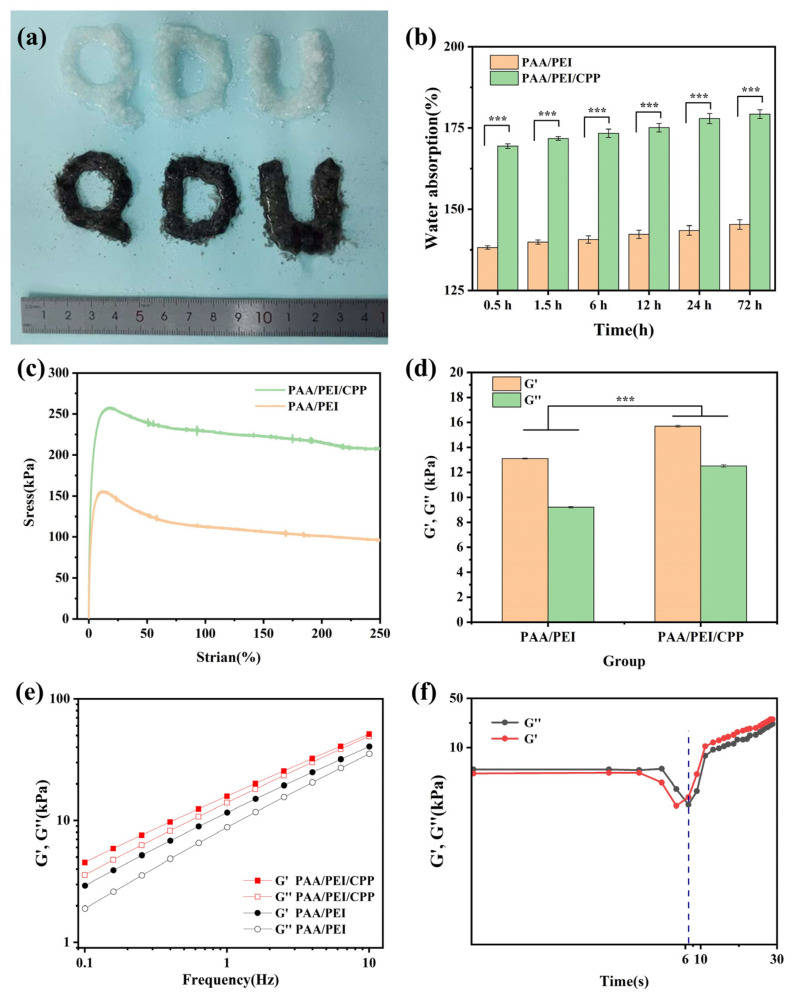
(**a**) The white PAA/PEI powder and black PAA/PEI/CPP powder after adding deionized water. (**b**) Water absorption rate (*** *p* < 0.001). (**c**) Mechanical performance. (**d**) Storage modulus (G′) and loss modulus (G″) of PAA/PEI hydrogel and PAA/PEI/CPP gel (*** *p* < 0.001). (**e**) Storage modulus (G′) and loss modulus (G″) as a function of frequency in PAA/PEI hydrogel and PAA/PEI/CPP gel. (**f**) PAA/PEI/CPP powder gelling time after water absorption (time-dependent curves of storage modulus G′ and loss modulus G″ measured using a rheometer at 37 °C).

As shown in Figure 2a, the powders were arranged in the shape of the letters “QDU”. After adding drops of deionized water, they gelled quickly. The CPP in the gel had a great influence on the water absorption rate of PAA/PEI (*** *p* < 0.001) and its mechanical properties. As shown in Figure 2b, with the prolongation of time, the water absorption rate of the PAA/PEI/CPP powder increased from 169.4 ± 0.73% to 179.3 ± 1.35%. After absorption, the maximum stress of the PAA/PEI/CPP gel reached 257.5 kPa, and then it began to decline (Figure 2c). In a rheological dynamic test, the storage modulus (G′) is the ratio of the energy stored by a material to the amplitude of its strain, reflecting the elastic recovery energy of the material under periodic loading. The loss modulus (G″) is the ratio of the energy dissipated by a material to its strain amplitude, reflecting the ability of the material to recover the energy dissipated during deformation. The storage modulus and loss modulus of the PAA/PEI/CPP gel also increased gradually with the addition of the CPP (Figure 2d), indicating that the larger the elastic recovery ability of the gel, the more energy consumed by recovery deformation (*** *p* < 0.001). In addition, G′ and G″ were dependent on frequency and represented a linear distribution (Figure 2e), suggesting that energy storage was relatively higher than energy loss in the two groups.

A rapid gelation time contributes to the acceleration of the hemostasis of wounds. Anticoagulant rabbit blood was added to the PAA/PEI/CPP powder to examine the gel time. As shown in Figure 2f, it was found that within 0–6 s, the G′ of the system was less than G″, indicating that the system tended to be in a viscous liquid state at this time. Within 6–8 s, the two modulus curves intersected, indicating that the system changed from a viscoelastic liquid to a viscoelastic solid, and gel gradually formed. After 6 s, G′ was always greater than G″, and the curve gradually rose steadily, indicating that the gel system was continuously absorbing water in the blood. The above experiments confirmed that the PAA/PEI/CPP powder could quickly absorb water to form gel, which is crucial for the hemostasis and sealing of wounds.

### 3.2. Antibacterial and Biocompatibility Investigations

**Figure 3:**
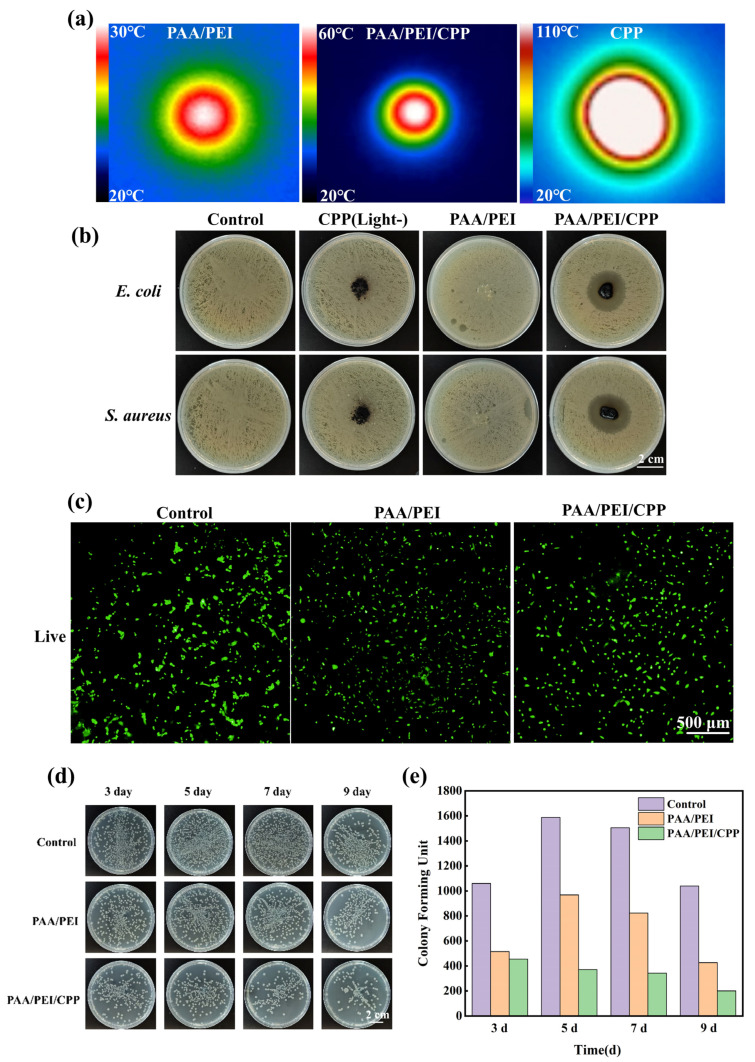
(**a**) Temperature variations in different groups after laser irradiation for 3 min. (**b**) Images of bacteriostatic effects against *E. coli* and *S. aureus* in different groups. (**c**) Live cell staining of mouse embryonic fibroblasts after 24 h culture in different groups. (**d**) Bacterial growth in different periods. (**e**) Colony forming unit in different periods.

The photothermal characteristics of the PAA/PEI/CPP powder under light were evaluated using a thermal imaging apparatus. Figure 3a shows that the maximum central temperature of the PAA/PEI powder was recorded at 30 °C after 3 min of irradiation with 808 nm light, whereas the central temperature of the PAA/PEI/CPP powder reached 60 °C, proving that, after CPP incorporation, the hydrogel powder was endowed with photothermal properties. Furthermore, research has shown that a high temperature is lethal to most bacteria, demonstrating potential antibacterial attributes of the PAA/PEI/CPP powder. For further validation, we conducted experimental investigations on the bacteriostatic effects of the CPP, PAA/PEI powder, and PAA/PEI/CPP powder against *E. coli* and *S. aureus* (Figure 3b). The bacterial areas of the culture media in the control group and PAA/PEI powder group both achieved close to 100% after 12 h of culture. Significantly, the formation of antibacterial rings against *E. coli* and *S. aureus* bacteria was observed in the presence of the PAA/PEI/CPP powder, confirming its potent antibacterial activity. In addition, no antibacterial ring appeared in the “CPP (Light-)” group (Figure 3b, pure CPP treated without laser irradiation) or in the PAA/PEI group, indicating that the pure CPP or PAA/PEI had no antibacterial activity. Under laser irradiation conditions, the central temperature of the CPP reached up to 110 °C (Figure 3a); this high temperature could melt the agar plate and even affect normal tissue or wound recovery. Therefore, the following observation rejected the pure CPP (Light-) group in Section 3.2.

To investigate the biocompatibility of the PAA/PEI/CPP powder, which is crucial for wound dressings, we conducted an in vitro culture of mouse embryonic fibroblasts with both the PAA/PEI and PAA/PEI/CPP powders, followed by cell staining after 24 h of culture. The survival of the mouse embryonic fibroblasts after 24 h of incubation with the PAA/PEI and PAA/PEI/CPP powders is depicted in Figure 3c. Notably, both of the two groups exhibited comparable cell viability to the control group, indicating that the PAA/PEI/CPP powder had no effect on the biological activity of cells.

After infection, wounds often heal slowly. The antibacterial activity of wound dressings could effectively inhibit bacterial infection and reduce the occurrence of wound complications. According to the data statistics in Figure 3d,e, the colony forming unit in the PAA/PEI and PAA/PEI/CPP groups was lower than that in the control group, indicating that the “material barriers” formed by the two groups could reduce infection. The bacterial count in the PAA/PEI/CPP group was the lowest, suggesting that it can induce bacterial death.

### 3.3. Coagulation Outcomes

**Figure 4:**
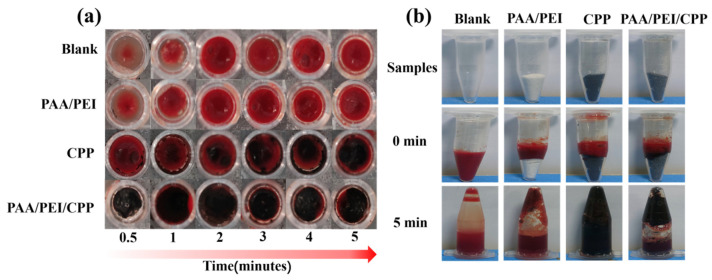
(**a**) Coagulation effect of various materials on a 96-well plate. (**b**) Image of blood clot formation in an in vitro coagulation experiment.

To study the coagulation effect of the PAA/PEI hydrogel combined with the CPP, we further conducted in vitro coagulation experiments. The coagulation effects of the PAA/PEI powder, CPP, and PAA/PEI/CPP powder are shown in Figure 4a. The blank group achieved the coagulation effect after 5 min. As for the PAA/PEI powder, its coagulation was completed within 3~4 min, demonstrating excellent physical adhesion. The CPP only floated on the blood surface, and an unobvious coagulation effect was observed; this could be attributed to the hydrophobic properties of CPP. When PAA/PEI was combined with the CPP, the PAA/PEI/CPP powder achieved coagulation within 2 min due to the adhesion and hydrophilicity of the PAA/PEI powder, indicating the occurrence of a synergistic coagulation effect of the PAA/PEI powder and CPP.

Figure 4b allows for the hemostatic effect of the PAA/PEI/CPP powder to be observed in a more intuitive way. The PAA/PEI powder, CPP, and PAA/PEI/CPP powder were placed in a centrifuge tube, and treated rabbit blood was added. After standing for 5 min, PBS buffer was added, and the tube was reversed. Only a few blood clots appeared in the control group after 5 min, demonstrating a weak hemostatic effect of the CPP group. The hydrophobic CPP mixed with the blood and became a suspension, so it had no hemostatic effect. The PAA/PEI powder formed a more apparent blood clot on top of the centrifuge tube because of its excellent adhesion. The largest blood clot volume formed on top of the PAA/PEI/CPP powder centrifuge tube, demonstrating that the PAA/PEI powder and CPP had a synergistic hemostatic effect after being combined, which effectively improved coagulation efficiency.

### 3.4. Animal Experimental and Histological Analyses

**Figure 5:**
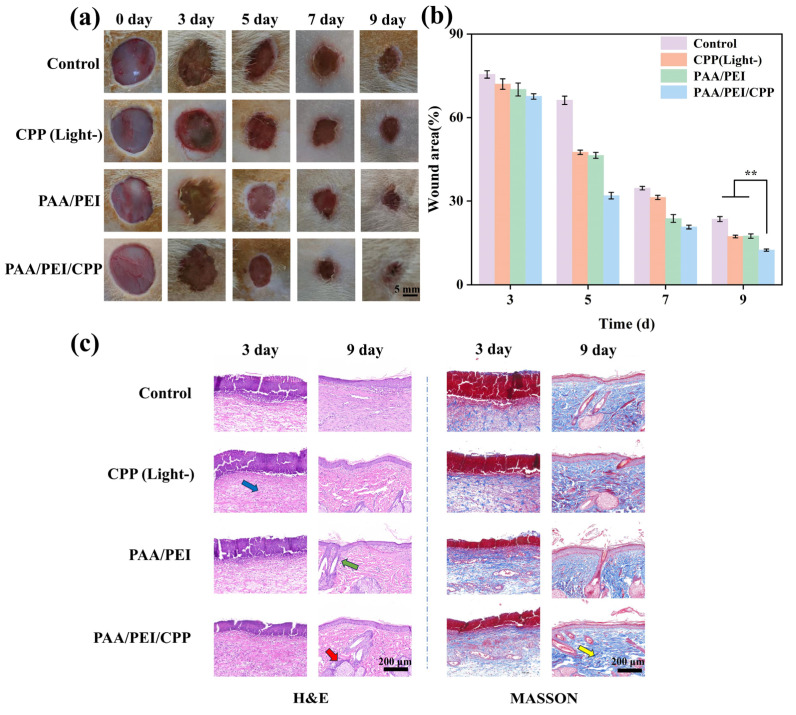
(**a**) Wound recovery status in various periods. (**b**) Wound area percentage in various periods (** *p* < 0.01). (**c**) H&E and Masson staining of each group in different periods (the blue arrow indicates inflammatory cells, the green arrow indicates hair follicles, the red arrow indicates blood vessels, and the yellow arrow indicates collagen fibers).

We conducted an experiment by establishing a rat back wound model to determine whether the PAA/PEI/CPP gel could promote wound healing (Figure 5a). It was evident in all groups that, as time increased, the wound area gradually decreased. Notably, on the 9th day, the control group exhibited the largest wound size, while both the CPP (Light-) and PAA/PEI powder groups showed similar wound recovery conditions. However, the PAA/PEI/CPP powder group exhibited superior wound healing due to its excellent adhesion properties, as well as antibacterial and anti-inflammatory functions. This was because the carbonized plant powder in the CPP (Light-) group was prone to detachment, resulting in inadequate efficacy on the wound and poor wound recovery. Although no detachment occurred in the PAA/PEI powder group, its overall wound recovery was unoptimistic, implying that it lacked efficacy in promoting wound healing. Therefore, only the application of the PAA/PEI/CPP powder resulted in significant improvement in wound recovery outcomes. To further validate this effect quantitatively, we performed an analysis of wound area changes at different time points, as shown in Figure 5b. Remarkably, on the 5th and 7th days, noticeable improvements were observed, specifically in the PAA/PEI/CPP powder-treated wounds. On the 9th day, only approximately 12% of the wound area remained, thus confirming its pronounced ability to promote effective wound healing.

To conduct a more comprehensive investigation into wound healing, H&E staining and Masson staining were performed on the tissue sections of the wounds (Figure 5c). H&E staining revealed that, after 9 days of treatment, there was a significant presence of dark blue neutrophils in the subcutaneous tissue of the control, CPP (Light-), and PAA/PEI powder groups, suggesting tissue inflammation. The PAA/PEI/CPP powder group exhibited the lowest neutrophil count relatively and only mild inflammation; this was attributed to the photothermal sterilization and excellent adhesion properties of the PAA/PEI/CPP powder, which maximized its anti-inflammatory efficacy. Masson staining mainly reflected the changes in the collagen fibers in the tissues during wound healing. Denser, dark blue collagen fibers in the tissues indicated better wound healing. After 9 days, the PAA/PEI/CPP powder group exhibited the highest density of collagen fibers and demonstrated angiogenesis, indicating its pronounced efficacy in promoting tissue regeneration. These findings demonstrate the multifaceted benefits of PAA/PEI/CPP powder, including wound healing promotion, sterilization, and anti-inflammatory properties.

## 4. Discussion

Due to the presence of hydrophilic groups, hydrogel can moisten dry and necrotic wounds, and it can surround nerve endings with a moist environment, thereby relieving pain. Hydrogel wound dressings initially only provided simple physical isolation and created a humid environment. However, with the continuous improvement of clinical requirements for wound repair, it has been found that rapid wound closure in the shortest time is crucial for wound healing [26]. Table 1 lists the features and wound closure rates of several composite traditional Chinese medicine gels/hydrogels. As a multifunctional gel, PAA/PEI/CPP possesses special functions in photothermal sterilization, rapid hemostasis, and anti-inflammation. Compared with the other five types of gels/hydrogels, a relatively high wound closure rate was observed on day 9, indicating its potential to become an important material for wound repair.

Skin damage caused by trauma usually leads to irregularly shaped wounds and slow healing. Composite hydrogels can be transformed from a solid state to a liquid state at body temperature, offering good shape adaptability and the ability to cover irregularly shaped wounds [32]. With enhanced mechanical properties, adhesive strength, and an injectable self-healing ability, many reported composite hydrogels can shorten the healing time of burn and irregular wounds, ranging from 12 days to 14 days [33,34]. As observed in Figure S2, directly applying CPP on the back of a tilted hand was likely to result in detachment, let alone on an irregular wound. Adhesion and gel time tests of the PAA/PEI/CPP powder confirmed that it could not only provide good adhesion but also absorb water within 6–8 s to induce gelatinization, which is crucial for the hemostasis and sealing of irregular wounds. The physical hemostatic ability of the PAA/PEI hydrogel solves the problem of CPP’s weak attachment.

Bacterial infection is also a significant factor during the wound healing process. The overuse of antibiotics may lead to the development of antibiotic resistance in pathogens. Therefore, hydrogels with photothermal antibacterial properties have become a hotspot in the antibacterial field. The photothermal sterilization of hydrogels combines the unique physical properties of hydrogels and the highly effective sterilization activities of photothermal conversion materials. Guo et al. [35] reported a composite antibacterial hydrogel, PDA-PAM/Mg^2+^, which could induce an elevated temperature of 21.3 °C after four on/off cycles and had high photothermal stability; the survival rate of *E. coli* was 7.06%, and that of *S. aureus* was 5.29%. Qi et al. [36] constructed a PDA@Ag biocompatible polysaccharide nanoparticle hydrogel. Its temperature could be raised from 25 °C to 50 °C within 3 min and reach 58.6 °C within 5 min. The hydrogel killed 99.9% of *E. coli* and 99.8% of *S. aureus*. In our experiment, the CPP actually had no antibacterial effect itself. After 3 min of irradiation, the central temperature of PAA/PEI/CPP reached 60 °C. This temperature enabled it to have a bactericidal effect on *E. coli* and *S. aureus*, thereby showing potential for reducing wound infection rates.

## 5. Conclusions

In this study, we innovatively combined traditional Chinese herbal CPP with hydrogel to prepare a PAA/PEI/CPP gel wound dressing, which could gel within 6–8 s, prevent CPP detachment, and firmly adhere to the surface of irregular wounds. Neither CPP nor PAA/PEI had antibacterial properties. Under laser irradiation for 3 min, the temperature of PAA/PEI/CPP was raised to 60 °C, and it was endowed with photothermal sterilization properties. It achieved a synergistic coagulation hemostatic effect within 2 min. Animal experimental and histological analyses showed its pronounced ability to repair 88% of the wound area by day 9, with an anti-inflammation function. The preparation methods and applications of integrating traditional Chinese medicine with hydrogel also provide new solutions for multifunctional wound dressings.

A shortcoming of this study is that the wound recovery observation time did not exceed 10 days, and thus, the long-term repair ability remains uncertain.

## Figures and Tables

**Table 1:** Features and wound closure rates of different gels/hydrogels.

Gel/Hydrogel Materials	Characteristics	Functions	Wound Closure Rate (%)	Reference
Chitosan and puerarin	Injectable and self-healing	Promoting diabetic wound healing and inhibiting inflammation	65% on day 7	[27]
Chitosan and puerarin	Inspiring mechanical force to promote self-assembly	Antibacterial and immune regulation	90% on day 14	[28]
Gelatinized starch, aloe vera, and berberine	Microneedles	Antibacterial, anti-inflammatory, and fibroblast growth-promoting effects	82% on day 7	[29]
Puerarin and silk fibroin induced by Ga ions	Dual nanofibrillar network structure	Sustainable retention, biocompatible, and anti-inflammation	100% on day 10	[30]
Scutellaria baicalensis extract, sesbania gum, and carboxymethyl chitosan	Oxidation of functional groups and chemical bonding	Promoting epidermal regeneration and collagen deposition	80% on day 7	[31]
PAA/PEI/CPP	Gelling within 6–8 s and quickly formed	Photothermal sterilization, rapid hemostasis, and anti-inflammation	88% on day 9	This work

## Data Availability

The original contributions presented in this study are included in the article/Supplementary Materials, further inquiries can be directed to the corresponding authors.

## Supplementary Materials

The following supporting information can be downloaded at: https://www.mdpi.com/article/10.3390/nano14241992/s1, Figure S1: (a) Adhesion test of PAA/PEI/CPP gel powder between two glass plates. (b) Adhesion test of PAA/PEI/CPP gel powder between glass and pig skin, Figure S2: (a) CPP is placed on the tilted back of the hand. (b) Water droplets falling onto CPP. (c) The vast majority of CPP are washed away.
