# Supplementary material for: Carbonized Plant Powder Gel for Rapid Hemostasis and Sterilization in Regard to Irregular Wounds
Source: Nanomaterials (Basel). 2024 Dec 12;14(24):1992. doi: 10.3390/nano14241992 (PMC11728490; doi:10.3390/nano14241992)
Supplement: Supplementary file 1 [file nanomaterials-14-01992-s001.zip › nanomaterials-3266968-supplementary.pdf]

# Supporting Information

# Carbonized Plant Powder Gel for Rapid Hemostasis and Sterilization in Regard to Irregular Wounds

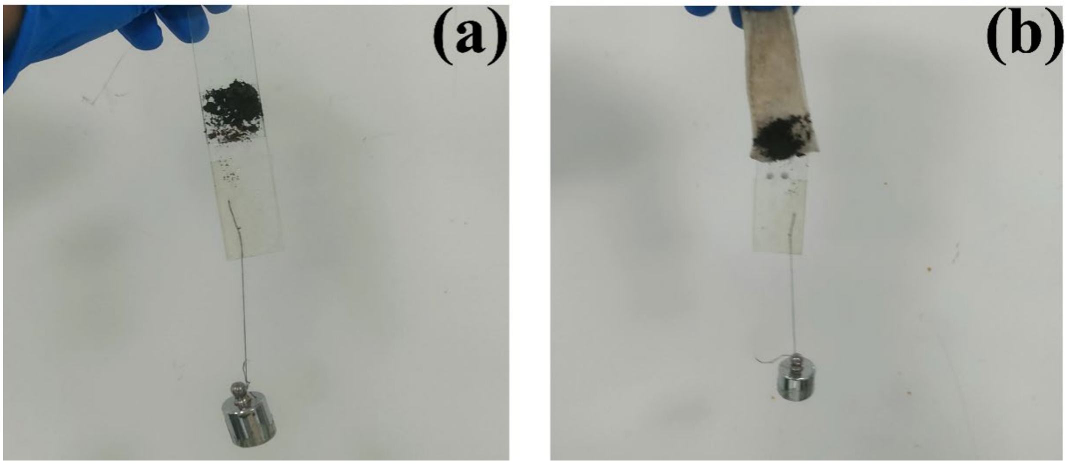


**Figure S1.** (**a**) Adhesion test of PAA/PEI/CPP gel powder between two glass plates. (**b**) Adhesion test of PAA/PEI/CPP gel powder between glass and pig skin.

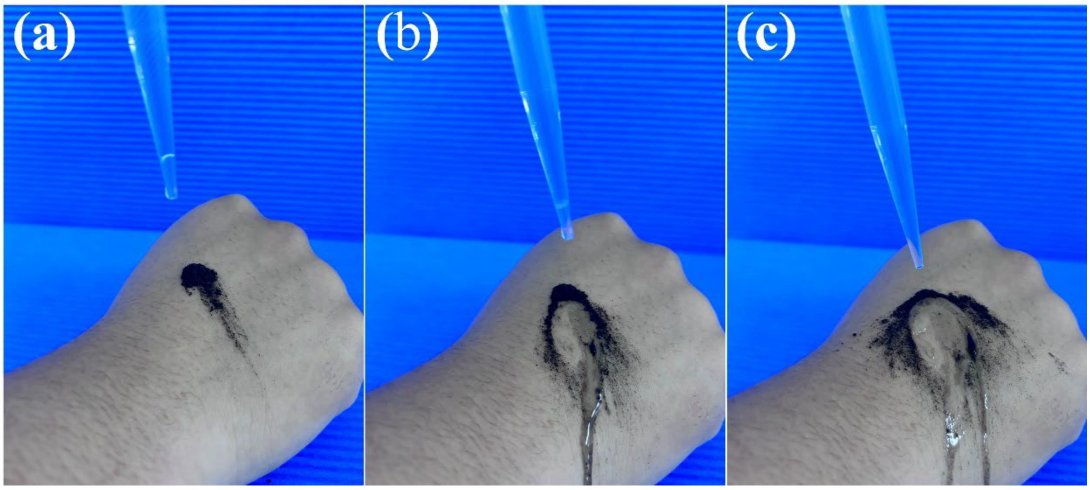


**Figure S2.** (**a**) CPP is placed on the tilted back of the hand. (**b**) Water droplets falling onto CPP. (**c**) The vast majority of CPP are washed away.
